# Fundamentals and applications of photocatalytic CO_2_ methanation

**DOI:** 10.1038/s41467-019-10996-2

**Published:** 2019-07-18

**Authors:** Ulrich Ulmer, Thomas Dingle, Paul N. Duchesne, Robert H. Morris, Alexandra Tavasoli, Thomas Wood, Geoffrey A. Ozin

**Affiliations:** 1 Department of Chemistry, University of Toronto, 80 Saint George Street, Toronto, ON M5S 3H6 Canada; 2 Department of Material Science and Engineering, University of Toronto, 184 College Street, Toronto, ON M5S 3E4 Canada

**Keywords:** Photocatalysis, Chemical engineering, Energy, Green chemistry, Materials chemistry

## Abstract

The extraction and combustion of fossil natural gas, consisting primarily of methane, generates vast amounts of greenhouse gases that contribute to climate change. However, as a result of recent research efforts, “solar methane” can now be produced through the photocatalytic conversion of carbon dioxide and water to methane and oxygen. This approach could play an integral role in realizing a sustainable energy economy by closing the carbon cycle and enabling the efficient storage and transportation of intermittent solar energy within the chemical bonds of methane molecules. In this article, we explore the latest research and development activities involving the light-assisted conversion of carbon dioxide to methane.

## Abstract (web summary)

While natural gas and fossil fuels power human activities, increasing concerns over fuel reserves and environmental impacts require finding alternative, renewable resources. Here, authors review the fundamental science and progress on solar-powered conversion of carbon dioxide to methane.

## Introduction

The combustion of natural gas (NG), which is used for heating, electricity generation and as a chemical feedstock, accounts for 20 wt% of global CO_2_ emissions^1^. While demand for NG is predicted to rise in the coming decades, known fossil fuel reserves are projected to last for only another 60 years^2^. Thus, a non-fossil-based, sustainable source of methane (CH_4_), the main component of NG, is needed in order to satisfy the growing demand for this important fuel and chemical feedstock while simultaneously reducing the environmental impact of its use.

Sustainable production of “synthetic” natural gas (SNG) is possible through the conversion of CO_2_ and water (H_2_O) into CH_4_ and oxygen (O_2_) using renewable energy such as sunlight. This reaction proceeds as follows:

1 $${\mathrm{{CO}}}_2 + 2{\mathrm{{H}}}_2{\mathrm{{O}}} \to {\mathrm{{CH}}}_4 + 2{\mathrm{{O}}}_2$$

The advantages of this approach are threefold:

1. The infrastructure required for the storage, distribution, and use of (S)NG is already established and readily available.
2. The high energy density of SNG makes it an efficient storage medium for excess renewable energy.
3. The high abundance and relatively low cost of CO_2_ and H_2_O feedstocks make SNG a significantly value-added product.

SNG production is already carried out through power-to-gas (PtG) technology. Solar PtG employs photovoltaic cells to electrolyze water and generate hydrogen (H_2_), which is subsequently reacted with CO_2_ to form CH_4_ via heterogeneous catalysis or biocatalysis. The best-case solar-to-methane efficiency is 13.0% (22.5% for solar cells^3^; 90% for CO_2_ capture from flue gas^4,5^; 80% for water electrolysis^6^ and 80% for CO_2_ methanation^7^).

Technologies capable of storing solar energy in the chemical bonds of CH_4_ with greater efficiency, relative to the aforementioned PtG method, are the subject of ongoing research^8^. In this review, we summarize the most recent such scientific discoveries that are paving the way towards a fully realized solar CO_2_-to-CH_4_ process. Here, the focus lies on the light-assisted CO_2_ methanation reaction. In principle, sunlight can also be incorporated into methanation processes by utilizing photocatalytic^9,10^ or solar thermal^11^ water splitting to produce the H_2_ consumed during CO_2_ methanation. The existing scientific literature^9–11^ summarizes the concepts and research activities in these fields.

**Fig. 1:**
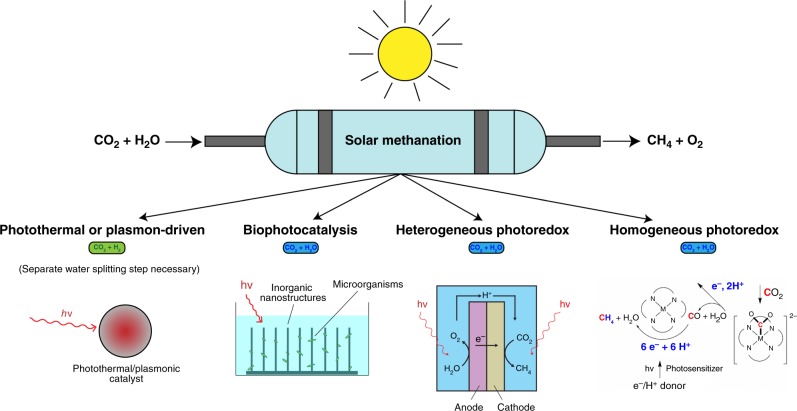
Schematic depiction of the solar methanation process and the various methods covered in this review paper. This includes the four main catalysis methods that are discussed: Photothermal or plasmon-driven, biophotocatalysis (hybrid bio-inorganic), heterogeneous photoredox and homogeneous photoredox

Here, though, we begin by reviewing the reaction energetics for the conversion of CO_2_ into CH_4_, then dive into a short summary of the state-of-the-art in current CO_2_ methanation systems. The most recent developments regarding the various solar methanation schemes covered in this review are discussed thereafter. The article structure is illustrated graphically in Fig. 1.

We first consider “photothermal” methods in which photons generate high catalyst temperatures^12,13^, thereby driving the CO_2_ methanation reaction. “Biophotocatalytic” methods are then discussed; these systems use microorganisms, or a combination of inorganic materials and microorganisms, to catalyze CO_2_ methanation. Finally, the latest developments regarding “photoredox” methanation systems are presented. This section is further subdivided into heterogeneous and homogeneous categories. These systems enable CO_2_ methanation via light-driven, photoelectrochemical reactions.

Under the IUPAC definition of photocatalysis^12^, all solar methanation schemes discussed herein are of photocatalytic nature, as they exhibit light absorption to achieve an excited state (“photoexcitation”), followed by interaction between the excited photocatalyst and reactant molecules to create products. While the light absorbed is restricted to ultraviolet, visible, and infrared wavelengths^12^, the excited state is not limited to electronic transitions and can include plasmonic, vibrational, or rotational excitations^12^.

## Reaction energetics

Thermodynamically, the reaction of CO_2_ and H_2_O to form CH_4_ and O_2_ (Eq. (1)) is endergonic with a large positive change of Gibbs energy ($\Delta G_{298\,{\mathrm{{K}}}}^0$ = 818 kJ mol^−1^ or 1.037 V_NHE_) and therefore does not occur spontaneously under standard conditions. If this source of energy is sunlight, the reaction is described as “artificial photosynthesis”, due to its conceptual analogy to “natural photosynthesis”, the process by which certain organisms synthesize complex organic molecules from CO_2_ using light^12^.

Theoretically, if both the reactants and products are at their respective thermodynamic standard states when entering and leaving the solar methanation reaction, then the minimum energy gap that has to be overcome is the energy difference between them (i.e., 818 kJ mol^−1^). In reality, though, this situation is more complex. When designing artificial photosynthesis reactions, such as solar methanation, it is useful to consider natural photosynthesis for inspiration. The reaction pathway for natural photosynthesis can be conceptually simplified into two separate reaction steps^14^: (1) light-driven splitting of water into “reducing equivalents” (i.e., [H], such as reduced nicotinamide adenine dinucleotide phosphate (NADPH)) and O_2_, and (2) CO_2_ conversion into glucose:

2 $$12{\mathrm{{H}}}_2{\mathrm{{O}}}\mathop { \to }\limits^{hv} 24\left[{\mathrm{{ H }}}\right] + 6{\mathrm{{O}}}_2$$

3 $$6{\mathrm{{CO}}}_2 + 24 \left[ {\mathrm{{H}}} \right] \to {\mathrm{{C}}}_6{\mathrm{{H}}}_{12}{\mathrm{{O}}}_6 + 6{\mathrm{{H}}}_2{\mathrm{{O}}}$$

4 $$6{\mathrm{{CO}}}_2 + 6{\mathrm{{H}}}_2{\mathrm{{O}}}\mathop { \to }\limits^{hv} {\mathrm{{C}}}_6{\mathrm{{H}}}_{12}{\mathrm{{O}}}_6 + 6{\mathrm{{O}}}_2;{\mathrm{\Delta }}G_{298\,{\mathrm{{K}}}}^0 = 2880\,{\mathrm{kJ}}\,{\mathrm{mol}}^{ - 1}$$

The majority of the energy required for photosynthesis is consumed by the water splitting step. NADPH, H^+^, and adenosine triphosphate (ATP) are then produced through a sequence of e^−^- and H^+^-transfer steps. These chemicals serve as e^−^-transfer, H^+^-transfer, and energy-transfer shuttles for the CO_2_ conversion reaction, which is separated from the water splitting reaction both spatially and temporally.

**Fig. 2:**
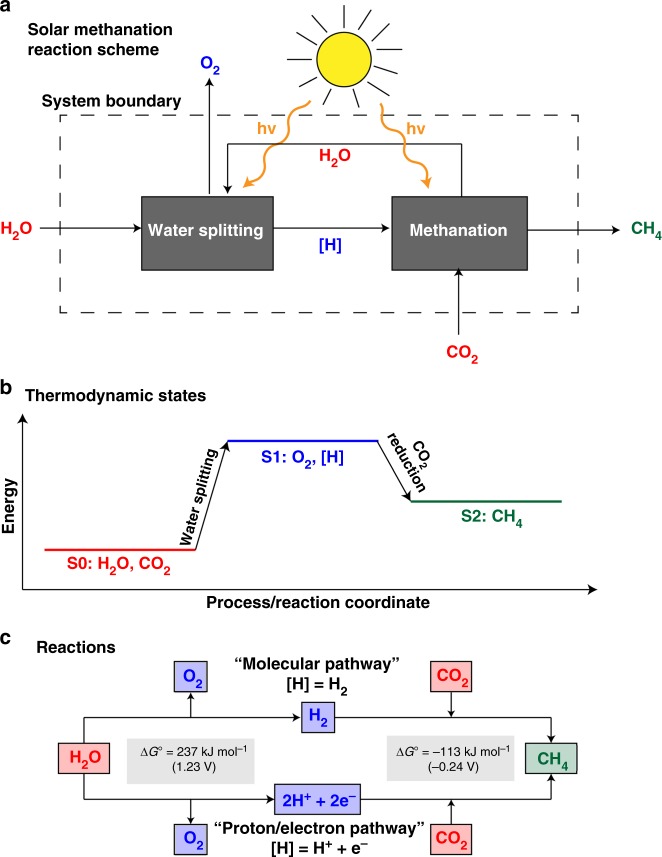
Graphical representation of solar methanation reaction schemes and energetics. **a** In a first reaction step, water is split into reducing equivalents [H] and O_2_. [H] can be molecular H_2_ or a H^+^/e^−^ pair. If [H] is molecular H_2_, then CO_2_ is reduced to CH_4_ via the “molecular pathway”. If [H] is a H^+^/e^−^ pair, then CO_2_ is reduced to CH_4_ via the “proton/electron pathway”. **b** Graphical representation of the thermodynamic states of the solar methanation reaction. The energy level of the system is elevated from S0 to S1 during the water splitting step. Energy is released during CO_2_ reduction, reaching the energy level of the products, S2. **c** Reactions occurring during the solar methanation pathways shown in **a**. The “molecular” pathway represents the state-of-the-art of industrial methanation, where H_2_ is produced during water electrolysis, as discussed in the “State-of-the-art industrial CO_2_ methanation” section

This example of natural photosynthesis illustrates that the complex synthesis of organic molecules from CO_2_ and H_2_O is most easily achieved by separating the water splitting and CO_2_ reduction reaction steps. A similar approach can be taken when designing artificial, light-driven methanation reaction schemes, as depicted in Fig. 2a. The corresponding thermodynamic states of the reactants and (intermediate) products are represented in Fig. 2b.

In emulating natural photosynthesis, this artificial solar methanation scheme separates the water-splitting and CO_2_-reduction reactions, with both reactants initially at their lowest energetic states (S0). Water splitting is an energy-intensive, endergonic reaction and driving this reaction elevates the energy level of the system to its highest energetic state (S1). Energy in the form of sunlight, electricity or heat must be supplied to initiate this reaction. During this first transition from S0 to S1, a reducing equivalent, [*H*], is produced; depending on the reaction scheme, this can be either molecular H_2_ or two H^+^/e^−^ pairs (Fig. 2c). State-of-the-art industrial methanation (discussed in the next section) produces molecular H_2_ via an electrocatalytic water-splitting approach, as do both the photothermal/plasmonic and biophotocatalytic methods described in this review. This is called the “molecular pathway”. If the evolution of molecular H_2_ is inhibited, then the reducing equivalent is two H^+^/e^−^ pairs, the “proton/electron pathway”. This is the case for both the heterogeneous and homogeneous photoredox methanation systems.

Under standard conditions (1 bar, 298 K, pH = 0), molecular H_2_ is thermodynamically equivalent to 2H^+^ and 2e^−^. Hence, an identical thermodynamic gap of $\Delta G_{298K}^0$ = 237 kJ mol^−1^ (equivalent to 1.23 V_NHE_) must be overcome to produce either H_2_ or 2H^+^/e^−^.

The reducing equivalents formed during water splitting are subsequently consumed during CO_2_ methanation. The change in Gibbs energy for reducing CO_2_ to CH_4_ is negative. Hence, it is an exergonic reaction and proceeds spontaneously under standard conditions. As a result, the system energy decreases to S2, which lies between the initial S0 and S1 states.

## State-of-the-art industrial CO_2_ methanation

Currently, CO_2_ methanation is accomplished through heterogeneous catalytic or biocatalytic methanation (abbreviated HM and BM, respectively). In both approaches, CO_2_ methanation is performed via two successive steps. First, water is split electrolytically into molecular H_2_ and O_2_ using electricity derived from a renewable source (e.g., wind or solar). Next, H_2_ is used as a reducing agent to convert CO_2_ to CH_4_. The CO_2_ is typically obtained from stationary point sources, such as biogas or wastewater treatment plants^15^.

In HM, the reduction of CO_2_ to CH_4_ is performed using solid-phase metal catalysts, such as Ni, Ru, Rh, and Co, supported on metal oxides, such as Al_2_O_3_ or ZrO_2_^16^. Ni serves as the catalyst of choice in most methanation plants due to its high activity, good CH_4_ selectivity and low cost. Fluidized bed or fixed bed methanation reactors are usually preferred. Common operating temperatures are between 200 and 550 °C, with pressures ranging from 1 to 100 bar^7^.

In BM, CH_4_-producing microorganisms are applied as biocatalysts. CH_4_ and H_2_O are thus produced during the anaerobic reduction of CO_2_ by H_2_. This reaction occurs at 20–70 °C and 1–10 bar. The microorganisms are typically contained in a liquid fermentation broth within stirred tank or fixed bed reactors^7^.

The largest HM/BM plants are currently installed in Werlte, Germany (HM, 6 MW_el_ electric power input)^17^ and in Avodøre, Denmark (BM, 1 MW_el_ electric power input)^18^. The scientific literature summarizes the currently installed methanation projects^7,19^.

The main bottlenecks limiting the more widespread and economic operation of HM and BM methanation are:

1. *Cost of renewable hydrogen*: Water electrolysis is an energy-intensive process responsible for about 80% of the total capital and operational costs of a methanation plant^7^. Improvements in the economics of hydrogen production, or omission of the electrolysis step altogether, could significantly improve the economics of CO_2_ methanation.
2. *Purity of reactants*: Impurities present in feed gases may affect methanation catalyst performance, leading to the diminishment of both the rates and lifetime of catalysts. Catalysts applied in HM are more sensitive to impurities than the microorganisms employed in BM^19–21^. Thus, the feed gas typically has to be cleaned before entering a HM reactor, resulting in additional costs.
3. *Quality of products*: The properties of SNG must be similar to those of NG distributed in the grid. NG contains ~80% CH_4_, with the remainder consisting primarily of heavier hydrocarbons. The caloric value of NG is higher than that of pure CH_4_ and additional equipment and processing must be provided to adjust the gas composition (and caloric value) of SNG to meet the required standard^7^, again entailing additional steps and costs.
4. *Methanation reactor heat management and load flexibility*: The intermittent availability of renewable electricity means that hydrogen produced by renewably driven electrolysis cannot be supplied to the methanation reactor continuously. Therefore, dynamic operation of the methanation reactor is necessary, under which the reactor temperature changes drastically unless reactor heating and cooling can be quickly adapted. It is desirable to minimize reactor start-up and shutdown times to facilitate fast responses to load changes, thereby necessitating the use of efficient heating and cooling systems.

Overall, the incorporation of light could be useful for reducing or eliminating bottlenecks and challenges experienced during state-of-the-art industrial methanation. This will be discussed further in the “Industrial implications” paragraphs following the review of each photomethanation method.

## Photothermal and plasmon-driven methanation

In this approach to methanation, the illumination of the catalyst by light increases its local temperature via the photothermal effect, which then drives the methanation reaction. As photothermal or plasmon-driven CO_2_ methanation using H_2_O and CO_2_ remains to be definitively demonstrated, all photothermal catalysts discussed herein perform methanation using CO_2_ and H_2_ as reactants. A sustainable source of molecular H_2_ is necessary to fuel this reaction.

**Fig. 3:**
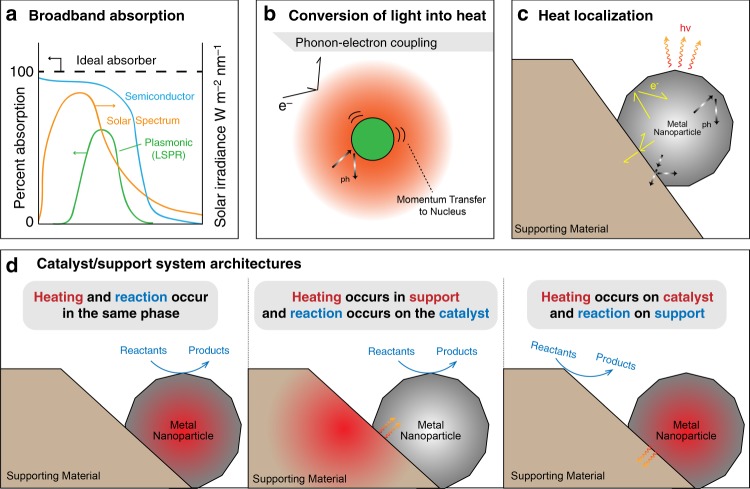
Key concepts and examples of photothermal methanation architectures. **a** Schematic representation of the light absorption spectra of semiconductors and plasmonic metals in comparison to the solar emission spectrum. **b** Light-to-heat conversion: photo-excited electrons (e^−^) interact with atomic nuclei, possibly generating phonons (ph). **c** Mechanisms of heat transfer within a particulate catalyst. Heat can be localized in catalyst nanoparticles by inhibiting phonon and electron transfer from the nanoparticle to its support. Structural defects, as well as phase boundaries between the catalyst and support phases, inhibit phonon and electron transfer and are hence beneficial for heat localization. Properties such as thermal and electronic conductivity, size and shape of the catalyst/support systems govern nanoscale heat transfer. **d** Selected potential photothermal catalyst architectures

Photothermal catalysis begins with the excitation of electrons via light absorption. Depending on the catalyst and the energy of the incident photon, this may involve interband, intraband, or plasmonic excitations of electrons^22–25^. Interband excitations occur between valence and conduction bands, intraband excitations occur to or from defect states within a band^22–24^ and plasmonic excitations involve a collective excitation of conduction band electrons^25^. “Antenna effects” may also increase the photon absorption cross-section of a plasmonic metal nanoparticle beyond its geometrical boundaries, thus enabling stronger light absorption^25^ (Fig. 3a).

Following electronic excitation, the captured energy must be converted into heat. Non-radiative electron relaxations, in which energy is transferred from excited electrons to adjacent atomic nuclei (Fig. 3b), are required to enable this conversion. Non-radiative energy transfer is dominated by processes such as electron–phonon scattering^26^, which is accomplished particularly well in plasmonic materials^27^.

When designing an effective photothermal catalyst, it must be ensured that the generated heat is localized to active catalytic sites driving the reaction. Depending on the catalyst architecture, this may mean heat is either transferred to or retained at catalytic sites^25^, and adequate heat transport strategies must be implemented that are effective at the nanoscale. Many catalysts employ a support material to enhance catalytic activity and stability, and the electrical and thermal conductivities of each component are important. Additionally, while they may present a barrier to heat transfer from the support, phase boundaries create an opportunity for thermal energy carriers to scatter back into the active catalyst material^28^, which can more effectively localize heat and thereby maximize the photothermal effect (Fig. 3c, d).

In addition to their usefulness as photothermal catalysts, plasmonic nanometals can initiate surface chemical reactions via “hot electron injection”, wherein the energy of localized surface plasmons excite charge carriers (electrons and holes) on the metal surface. These charge carriers are then transferred to an adsorbed reactant or intermediate and create excited states that facilitate chemical transformation of the adsorbed species^29^.

Nanoscale metal catalysts used for HM (such as Ni^30,31^, Ru^32–35^, Rh^36,37^, Fe^30,38^, Au^39^, and Pd^30,40^) are also often studied as photothermal catalysts, due to their strong broadband optical absorption. These catalysts may be supported on various materials to enhance their dispersion and stability, including Al_2_O_3_^30,31,37^, ZnO^39^, TiO_2_^30,36^, Nb_2_O_5_^40^, Si^32,33,35^, and metal-organic frameworks (MOFs)^38,41^.

Metal nanostructure morphologies, especially edges and corners, are known to enhance the local electric fields generated by oscillating plasmonic electrons, thereby amplifying light absorption. Furthermore, the lower coordination of atoms found at edge and corner sites, in combination with these enhanced electric fields, facilitates the injection of electrons into adsorbed reactants or intermediates, thereby modifying reaction pathways and rates^42–44^.

The excitation of plasmon resonance in Rh nanoparticles, for example, results in up to a seven-fold increase in selectivity for CH_4_ over CO, relative to thermal reaction conditions^37^. Theoretical simulations suggest that, in the thermocatalytic reaction, phonons activate intermediates during both CH_4_ and CO formation, resulting in comparable production rates of these two products. In the photocatalytic reaction, however, hot electrons selectively transfer to CH_4_ intermediates, reducing the activation energy of CH_4_ formation and increasing its production rate^37^.

The choice of the supporting material can greatly influence light absorption by the catalyst/support system. Traditional methanation catalyst supports (e.g., Al_2_O_3_ and TiO_2_) exhibit high specific surface areas and CO_2_ adsorption capacities, which are beneficial for catalysis; however, these supports are not optimal for light-harvesting. To improve this situation, researchers have studied highly light-absorptive support materials, such as vertically aligned Si nanowires^33,45^ and inverse opal Si photonic crystals^32,35^. The minimal reflection losses and strong broadband absorption made possible using these materials are instrumental in enhancing CO_2_ methanation rates^32,33,45^. For example, photothermal methanation rates for RuO_2_ dispersed on inverse opal Si photonic crystals (denoted as RuO_2_/i–Si–o), were enhanced relative to RuO_2_ deposited on a Si wafer. No photocatalytic effects were observed and the enhancement was attributed to increased temperatures resulting from improved light harvesting by the i–Si–o support. Density functional theory (DFT) was also used to study the reaction mechanism, suggesting that methanation was initiated via the interaction of H_2_ with oxygen atoms of RuO_2_ to form hydroxyl groups, which then interacted with CO_2_ to ultimately form CH_4_^32^.

Given that illumination of photothermal catalysts can initiate both photochemical and thermochemical reactions, knowing the respective contributions of each to reaction rates, selectivities and turn-over numbers is essential for understanding reaction mechanisms and designing improved catalysts. Fortunately, these reaction types can be differentiated based on their response to light. Plasmonically initiated reactions exhibit a super-linear (“power law”) dependence on light intensity (i.e., rate∝intensity^*n*^)^46^ and are characterized by a positive relationship between quantum efficiency and photon flux/temperature^43^. Thus, unlike traditional semiconductor-based photocatalysis, wherein quantum efficiency decreases with temperature, heat and light work synergistically in plasmonic reactions: increased temperature yields increased efficiency^36,42,43^.

Scientific challenges associated with the design and testing of photothermal methanation catalysts are: (1) correlating morphology, size, and composition of the photothermal catalyst/support system with its light-harvesting and catalytic properties; (2) identifying effects of light intensity and spectral distribution on the light-harvesting properties, quantum efficiency, catalytic rate/selectivity and temperature evolution, and distribution within the catalyst bed; and (3) distinguishing between thermal and photocatalytic effects on catalytic activity and selectivity.

Industrial implications: Illumination of a photothermal CO_2_ methanation catalyst could benefit an industrial methanation operation in a few key capacities. First, the photothermal effect could be used to heat a reactor system. Reactor heating is important during a cold start of the methanation reaction. Traditional methanation reactor systems accomplish this through the use of a heating jacket or other heat exchanger apparatus^7^. The advantage of photothermal systems is that high local temperatures can be generated very close to the catalytic site, thereby reducing heat transfer distances as compared to traditional heating methods and enabling faster start-up times and improved load flexibility.

Second, the introduction of light into a methanation reactor could increase the effective reaction rate relative to the thermocatalytic reaction through photocatalytic or plasmonic effects. This could increase the achievable throughput of reactants for a given catalyst mass or enable a smaller reactor to achieve the same throughput.

Third, though not a direct application of light to the CO_2_ methanation reaction, plasmonic photocatalysts could be used to adjust the caloric value of SNG by producing higher-value chemicals such as ethane or other higher hydrocarbons. These products can be targeted during the photothermal CO_2_ hydrogenation reaction^47^ or, alternatively, photocatalytic dehydrogenative coupling can be applied to convert CH_4_ into higher hydrocarbons^48–51^. A recent study has shown that the dehydrogenative coupling reaction can be initiated using a plasmonic photocatalyst composed of Au nanoparticles deposited on ZnO nanosheets^49^. According to experimental and theoretical methods, this reaction is induced by the electron transfer between the photoexcited ZnO nanosheets and a surface-adsorbed CH_4_ molecule. The reported quantum efficiency was 0.08%, which is comparable to that of natural photosynthesis^49^. Plasmonic photocatalysis thus represents a compelling method of tuning product selectivity and forming high-value products at milder temperatures than those used in common thermocatalytic reactions.

## Biophotocatalytic methanation

In this method, methanogens, “methane-producing” microbes, or hybrid systems composed of inorganic materials coupled with microbes convert CO_2_ and H_2_O into CH_4_ via biophotocatalytic reactions. The inorganic materials produce molecular H_2_, which is fed to the microorganisms to fuel the CO_2_ reduction reaction.

Methanogens live in anaerobic environments on Earth and release around one billion (10^9^) tonnes of the gas every year^52^. Since the reaction of H_2_ with CO_2_ is an exergonic process, it can be utilized by the microbes as a source of energy, which is ultimately stored in molecules such as ATP. While photosynthesis is not required for this form of CO_2_ metabolism, the generation of dihydrogen, or other reducing equivalents, using solar energy can be linked to CH_4_ formation through the use of bioreactors containing methanogens^52^.

**Fig. 4:**
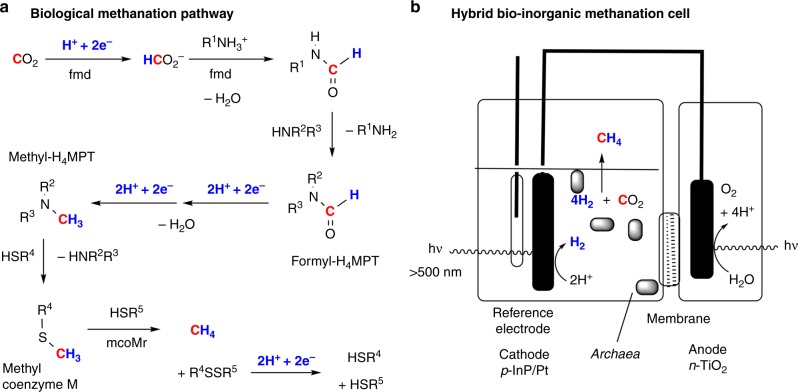
Biomethanation reaction systems. **a** A simplified scheme describing the methanation of CO_2_ catalyzed by archaea. Reproduced from ref. ^97^ (Copyright [2002], Elsevier)^97^. **b** A hybrid system for photomethanation utilizing CO_2_-metabolizing archaea in the cathode compartment as adapted with permission from ref. ^58^

Several of the enzymes involved in this CO_2_ metabolism have been structurally and chemically characterized. A key enzyme for the reduction of carbon dioxide is formylmethanofuran dehydrogenase (fmd)^53^. Here CO_2_ receives a hydride equivalent (H^+^ and 2e^−^) from a sulfur-ligated Mo^4+^ or W^4+^ active site of fmd to produce formate (Fig. 4a). The electrons for the hydride equivalent are carried from the dihydrogen-oxidizing Fe–Ni active site of a nearby hydrogenase to the CO_2_-reducing active site via a series of iron–sulfur clusters in the fmd protein.

The formate product then reacts with a primary amine, methanofuran (R^1^NH_2_ in Fig. 4a where R^1^ NH_2_ refers to enzyme cofactors with 2-aminomethylfuran linked to a substituted phenoxy group), at a dimeric Zn^2+^ site to produce the N-formyl compound R^1^NHCHO (formylmethanofuran). Next, a series of reactions converts the N-formyl compound into an N-methyl compound at successive enzymes upon receiving four electrons (and four protons) from Ni–Fe hydrogenases. The final CH_4_ evolution step requires that the methyl group first be transferred from nitrogen to sulfur in the form of the thioether compound R^4^SMe (methyl coenzyme M). The thioether and a thiol R^5^SH (coenzyme B) are then combined at the tetrapyrrole-ligated nickel active site of methyl coenzyme M reductase (mcoMr) to produce CH_4_ and the disulfide R^4^SSR^5^ in an exergonic process (−45 kJ mol^−1^). The reduction of the disulfide to the starting thiols completes the catalytic cycle, consuming the last two electrons of the eight electrons required for the reduction of CO_2_ to CH_4_.

This chemistry has evolutionary roots in the earliest forms of life^54^. Methanogens of the “archaea” class evolved to utilize transition metals (e.g., Fe, Ni, Zn, and Mo) that were abundant in the anoxic environment of the early Earth. Consequently, they are oxygen-sensitive, as are the hydrogenase enzymes that link the H_2_ oxidation and CO_2_ reduction reactions. There are a number of challenges facing the engineering of bioreactors to make this link function efficiently^55^. In addition to issues pertaining to the growth medium and the ultimate fate of deceased archaea cells, the low solubility of H_2_ in water and the slow transport of H_2_ and CO_2_ into water are key limitations. A variety of bioreactor designs have been proposed to overcome this mass transfer problem, which have been discussed in the scientific literature^7,56^.

There are opportunities for improvement by incorporating inorganic components into such biological reactors^57^. Hybrid systems that place the archaea in the cathode compartment of a two-compartment electrolysis cell have recently been described (Fig. 4b)^58^. Using a platinum cathode, 110 mL of methane was produced over 7 days with a Faradaic efficiency of up to 86% at a low overpotential of 360 mV. The system could be made completely solar-powered by replacing the platinum cathode with a platinum-coated, photoactive *p*-InP cathode to feed the archaea with H_2_ and a photoactive n-TiO_2_ anode in the second compartment to provide the electrons for H_2_ production from water oxidation. This solar-powered system produced 1.8 mL CH_4_ after 3 days and required an anion exchange membrane between the compartments to minimize pH changes. The observed Faradaic efficiency of this system was higher (up to 74%) when blue light was filtered out from the cathode compartment, due to the sensitivity of the microbes to these wavelengths.

Cathodes supporting immobilized hydrogenases, combined with anodes functionalized with photosystem II, have also been successfully used to form a solar cell capable of generating H_2_^59^. Such a cathode might also be suitable for housing active methanogens, thereby enhancing the ability of archaea to convert CO_2_ into CH_4_. Furthermore, some methanogens may be able to make use of electrons directly from an electrode to reduce CO_2_ to CH_4_ thus eliminating the need for the electrolysis of water^59^.

Industrial implications: There is much current research into utilizing bacteria, both natural and engineered, for the selective production of higher hydrocarbons of value to the chemical industry^56,60^. The efficient and selective conversion of CO_2_ to CH_4_ by these microbes, despite the presence of impurities in the gas stream^7^, makes these biological approaches very appealing. High tolerance towards common impurities in flue gas and raw biogas has been demonstrated, including hydrogen sulfide, nitrogen oxides, ammonia, particulates, as well as partial tolerance for oxygen and ethanol^19^. It is yet to be determined whether the inorganic components, which play pivotal roles in hybrid bio-inorganic photocatalytic systems, can also retain their performance in the presence of feed gas impurities.

The aforementioned hybrid system was reported to have a solar-to-chemical efficiency of 10% and an electrical-to-chemical efficiency of 52%. This assumes efficiencies of 20% for solar-to-electrical conversion at the photovoltaic panel, 70% for electrical-to-hydrogen conversion and 86% for the conversion of CO_2_ to CH_4_. The scale-up of such bioreactors is the major obstacle to these technologies and presents challenges very different from those faced by more conventional homogeneous and heterogeneous catalytic processes. It is evident that further research is required before photobiocatalytic methanation becomes industrially viable.

In addition to the scientific and technological challenges associated with the biophotocatalytic approach to methanation, the use of large quantities of genetically modified, potentially harmful organisms requires adequate safety measures to prevent leakage of these microbes into the environment and ensure public acceptance for this technology.

## Photoredox methanation

Photoredox catalysis occurs because a catalyst in an electronically excited state can be more easily reduced and oxidized than one in its ground state^12^. Such photoexcited catalysts are used to reduce CO_2_ to CH_4_ via light-induced redox reactions. Herein, we distinguish between homogeneous and heterogeneous photoredox catalysis. In heterogeneous photoredox catalysis, semiconductor materials generate excited electronic states to drive heterogeneous redox reactions, such as CO_2_ methanation. In homogeneous photoredox catalysis, however, light absorption, reduction, and oxidation occur at several complexes in solution^12^.

In heterogeneous photoredox methanation, several requirements must be satisfied to make a photocatalytic system useful for CO_2_ methanation. A photon of sufficient energy must excite an electron from the valence band of a semiconductor to its conduction band to create an “electron–hole pair”. The photoelectrochemical methanation reaction can be described as half-reactions (Fig. 2c), wherein the photogenerated hole initiates the oxidation of water to O_2_ and H^+^, and the photoexcited electron activates the CO_2_ reduction reaction.

**Fig. 5:**
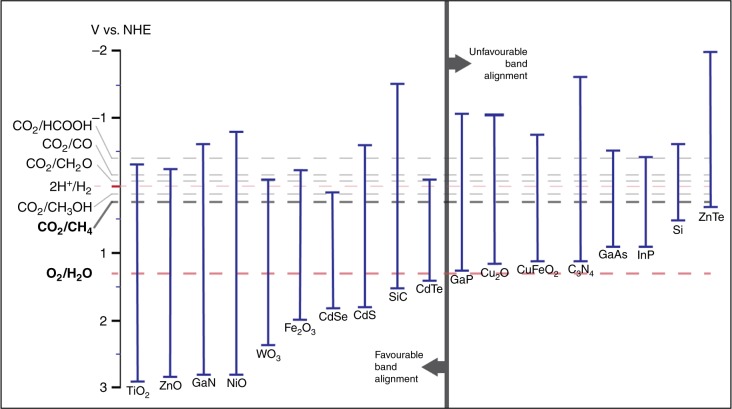
Band energy diagram of selected semiconductors. These materials are commonly used for photoelectrochemical water splitting and CO_2_ reduction. Redox potentials of key CO_2_ reduction reactions are also included. In principle, water splitting and CO_2_ reduction can take place on the same semiconductor material if the conduction band energy level is aligned with, or more negative than, the energy level of the targeted CO_2_ methanation reaction (−0.24 V_NHE_) and the valence band energy level is aligned with, or more positive than, the oxygen evolution reaction energy level (1.23 V_NHE_). This is indicated by the position of each material relative to the vertical bar dividing the figure. The materials exhibiting unfavorable band alignment are included in the figure, as they are commonly used as light-absorbers in photoelectrochemical cells^66, 98^—adapted from ref. ^66^—Published by Wiley-VCH; and ref. ^98^—Published by The Royal Society of Chemistry

**Fig. 6:**
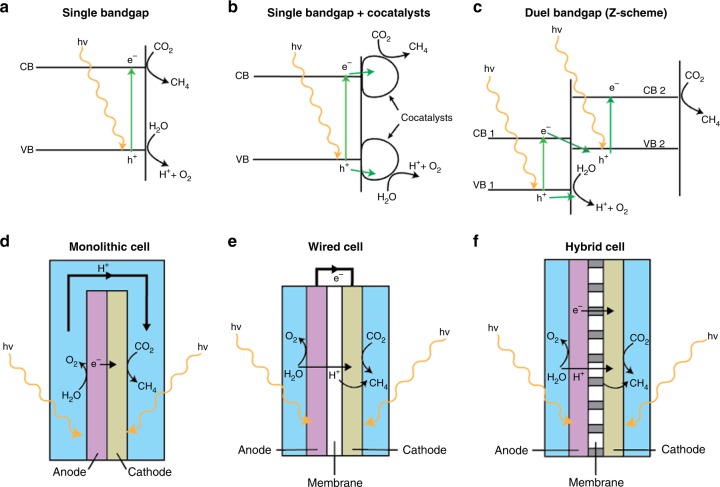
Band energy diagrams and device architectures of proposed heterogeneous photoredox methanation systems. **a** A single semiconductor photocatalyst that drives both the water oxidation and CO_2_ reduction reactions at its valence (VB) and conduction band (CB) sites. **b** A semiconductor photocatalyst with co-catalysts added to facilitate reduction and oxidation half-reactions. Electrons and holes can be transferred to the co-catalysts to initiate the associated half-reactions. **c** A Z-scheme consisting of two semiconductor photocatalysts, in which the water oxidation reaction is occurring at VB 1, and the CO_2_ reduction reaction occurs at CB 2. Electron transfer between the two semiconductors can be facilitated through the appropriate choice of semiconductors. Photoelectrochemical device architectures of (**d**) a monolithic device, in which protons and electrons are transferred from the anode to the cathode through an electrolyte or via conduction, respectively; (**e**) a wired device, in which protons and electrons are transferred from anode to cathode via a membrane and an external circuit, respectively; and (**f**) a photoelectrochemical cell, in which the anode and cathode are separated by a proton-conducting membrane with integrated electron-conducting material^99^. Adapted from ref. ^99^—Published by the Royal Society of Chemistry

The simplest case is a single semiconductor photocatalyst material, wherein both half-reactions occur at its surface. Figure 5 shows the band energies and redox potentials of a number of semiconductors. Theoretically, several of these materials could facilitate the water oxidation and CO_2_ reduction reactions (Fig. 6a shows the corresponding redox reaction taking place).

However, it is difficult to achieve the desired reaction using a single material. This is due to the complex, multi-step reaction mechanism requiring 8 protons and 8 electrons, which must be supplied sequentially at specific catalytic sites and intermediate stages of the reaction. To the best of our knowledge, there is no single material that has been confirmed, through ^13^CO_2_ labeling, to drive this entire process with light. Therefore, many researchers have focused their efforts on the development of composite materials^61–64^ and photoelectrochemical cells (PECs)^65–67^, in hopes of effectively combining light-absorbing and photocatalytically active materials.

As illustrated in Fig. 6b, co-catalysts can be added to tune product selectivity, reduce the activation barrier for redox reactions and facilitate the separation of charge carriers^68,69^. Composite heterogeneous photoredox catalysts are often composed of metals (e.g., Cu, Ru, or Re) coupled with semiconductors (e.g., p-Si, GaP, GaAs, GaN, or TiO_2_)^66^ or other metal co-catalysts like Au or Pt^62,65^. Other materials, such as lead-halide perovskite quantum dots supported on graphene oxide, have also been shown to photocatalytically reduce CO_2_ to CH_4_ and other products^70^; however, further tests are necessary to confirm the long-term stability of these materials in the presence of water. Grimes et al. have demonstrated that the addition of Pt and Cu onto TiO_2_ photocatalysts yielded CH_4_ under solar irradiation^71^. The addition of metal oxide co-catalysts, such as NiO and In_2_O_3_, to semiconductors (e.g., TiO_2_) has also been demonstrated to increase CH_4_ production^72^.

A common device architecture for CO_2_ methanation is the PEC, in which the photoredox steps occur on spatially separated materials. Figure 6c–f shows a number of layouts for PEC cells, in which the anode and cathode, on which the reduction and oxidation half-reactions occur, are separated from each other by e^−^-conducting and H^+^-conducting media. Much of PEC cell design is based around the spatial separation of photogenerated charge carriers, which has a substantial impact on the overall efficiency of such cells. Splitting the overall reaction into half-reactions allows PEC-based designs to use semiconductor materials with complementary bandgaps to improve efficiency by utilizing a broader range of the solar spectrum. This approach is commonly referred to as a “Z-scheme”, after the coupled photosystems used in plants^9^. Such systems have been studied as photoanodes for the water oxidation reaction, which is thermodynamically and kinetically demanding and represents the main bottleneck of the overall water-splitting reaction^73,74^.

The most complex of these architectures is the so-called “artificial leaf”. These systems aim to mimic the architecture of a leaf and, while having spatially separated photoredox reactions like PECs, feature micron-scale charge-carrier transport distances for both electrodes (rather than micron-scale for one and a larger length scale for the other). A promising strategy for the direct methanation of CO_2_ is using a 3D hierarchically structured perovskite titanate to mimic the structure of a natural leaf^75^.

Improvements, which are necessary to make heterogeneous photoredox methanation systems commercially more attractive are reaction product selectivity and cell-level efficiency. The electrochemical potentials of various CO_2_ reduction products are very close to that of the methanation half-reaction (Fig. 5), meaning that multiple reactions are likely to be occurring in parallel, resulting in low CH_4_ selectivity. This makes it rather challenging to achieve high Faradaic efficiency (FE), the percentage of charge carriers consumed in a particular reaction^65,66,76^.

Cell-level efficiency losses occur due to limitations on ionic conduction between electrodes. Nafion remains the best and most commonly used proton conductor in electrochemical systems^77^. Other media, including anion exchange membranes^78^, ionic liquids^79,80^, and bipolar membranes^81^, also offer interesting approaches to improving PEC systems, however, and should be further investigated with respect to photomethanation.

All PEC systems described in this review produce solar CH_4_ in a batch reactor mode. Additional research is necessary to enable the transition from batch to continuous flow PEC operation.

Homogeneous photoredox methanation involves the use of several molecular components. One molecule, the photosensitizer, usually but not necessarily a metal complex, acts as the light-absorbing agent. The absorbed energy is then either transferred to another metal ion complex called the photocatalyst, where the CO_2_ reduction reaction takes place, or it reacts with a sacrificial electron donor, whose electrons are transferred to the photocatalyst to perform the CO_2_ reduction reaction. These two complexes work synergistically to absorb light and convert dissolved CO_2_ into CH_4_ and other products.

Though few, reported catalyst complexes include iron^82^, cobalt^83^, and copper^84^ metal centers. In each case, a low-valent (i.e., Co^+^, Cu^+^, Fe^0^) metal complex bound to CO_2_ is postulated as part of the catalytic cycle. The cobalt and copper systems continue to convert CO to further-reduced products, including the 8-electron product methane (albeit with low Faradaic efficiency and only at electrode surfaces where copper nanoparticles are deposited^85^). Certain electrode-free photocatalytic systems utilize a photosensitizer to strip electrons from a sacrificial donor, such as triethylamine (TEA) to enable the reduction process.

**Fig. 7:**
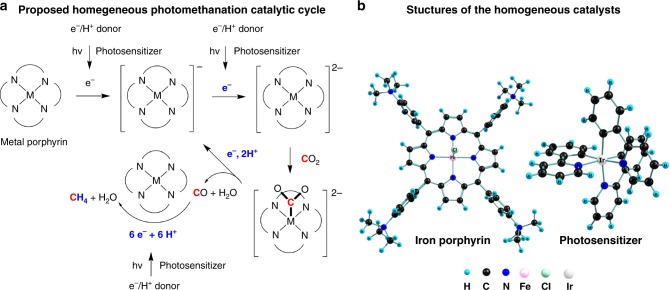
**a** A proposed sequence of steps in the photomethanation of CO_2_, as catalyzed by a metal porphyrin in solution. **b** Models of the catalysts and photosensitizers (some hydrogen atoms and counterions have been omitted for clarity). Adapted with permission from Nature Publishing Group: *Nature* **548**, 74–77, Visible-light-driven methane formation from CO_2_ with a molecular iron catalyst. Rao, H., Schmidt, L. C., Bonin, J. & Robert, M. (2017)

Recently, an iron tetraphenylporphyrin complex functionalized with trimethylammonio groups (Fig. 7) was incorporated into an electrode-free photocatalytic system utilizing visible light (>420 nm) for the methanation of CO_2_ at ambient pressure and temperature^82^. In this system, TEA is oxidized to an iminium radical that then decomposes to provide electrons at a negative electrochemical potential (less than −1.5 V_NHE_). These electrons are extracted from TEA by an iridium photosensitizer [Ir(ppy)_3_]^+^/[Ir(ppy)_3_)] (*E*^0^ = −1.7 V_NHE_, ppy = cyclometallated phenylpyridine) which then shuttles them to the CO_2_-activating iron center. The first CO_2_ reduction step is the conversion of CO_2_ to CO. Under optimized conditions, and in the presence of added trifluoroethanol, the photocatalytic oxidation of additional TEA molecules ([TEA]/[Fe] = 25,000) supplies two electrons to produce CO and another six electrons to reduce CO to CH_4_ with 82% selectivity, an estimated quantum yield of 0.18% and a turnover number of up to 159 for CH_4_ production along with up to 34 equivalents of H_2_ produced in side reactions. The origin of this methane product was verified, using GC–MS, by observing that only ^13^CH_4_ was produced from a ^13^CO_2_ feedstock. The iron-bound intermediates in this process remain unidentified, but the existence of an iron formyl (Fe–CHO) intermediate was postulated. It is worth pointing out that the alpha-/beta-elimination and hydride formation reactions leading to H_2_ evolution are inhibited by saturating the iron coordination sites, located *cis* to the reduced carbon species, with the four nitrogen atoms of the porphyrin ring. The iridium complex can also be replaced by a metal-free phenoxazine photosensitizer^86^. Several other metal-complex catalysts have been described in a review, including those based on Mn^+^, Re^+^, Fe^3+^, Ru^2+^, Co^+^, Ir^3+^, and Ni^2+^ for the photocatalytic reduction of CO_2_ to CO or formate, with triethanolamine or TEA used as the sacrificial electron donor^87^.

The advantage of homogeneous photoredox systems is their high product selectivity, while heterogeneous photoredox systems exhibit higher optical efficiencies and tunable optoelectronic properties as compared to homogeneous systems. Some novel approaches to CO_2_ photoredox catalysis aim to combine catalyst design principles from both heterogeneous and homogeneous photoredox systems, with the goal of developing photoredox catalysts that exhibit both the high optical efficiencies of heterogeneous semiconductor/plasmon-based light-harvesting systems with the superior product selectivity of homogeneous catalysts. MOFs are solid-state compounds consisting of metal ions or clusters coordinated to organic ligands, and are demonstrably suitable for such catalytic reactions^88^. Initial reports have shown the successful integration of known homogeneous CO_2_ photoreduction catalysts into the backbone of a solid-state framework and demonstrated the photocatalytic CO_2_ reduction activity of the obtained compounds^89,90^. This concept has been expanded to integrate plasmonic metal clusters^91^, metal oxides^92,93^, and photosensitizers^94^ into MOF architectures, thereby yielding new, high surface area, porous materials with tunable optoelectronic properties and catalytic activity. Reaction products were primarily formic acid, formate, and CO; however, this concept could potentially be applied to design photoredox catalysts with high selectivity for CH_4_.

Industrial implications: Various aspects of photoredox methanation make it advantageous for commercialization. First, the opportunity to avoid hydrogen production and storage steps makes photoredox systems a compelling way to increase the economic viability of industrial CO_2_ methanation. While the heterogeneous photoredox systems described in this review have effectively achieved the desired direct conversion of CO_2_ and H_2_O into CH_4_, their homogeneous counterparts require expensive sacrificial electron donors that limit their large-scale utility. Further research is required in order to identify homogeneous catalysts able to produce hydrocarbons directly from CO_2_ and H_2_O.

Second, photoredox systems operate at near-ambient pressure and temperature conditions, which are milder than the high-temperature, high-pressure conditions experienced in state-of-the-art heterogeneous catalytic methanation.

In addition to advances in materials development, further research is necessary to improve the designs of photoelectrochemical devices intended to reach technologically significant scales and improve solar-to-CH_4_ efficiencies. A solar-to-fuel conversion efficiency of 10% is regarded as the minimum value that PEC systems must achieve to exhibit similar or improved efficiency compared with state-of-the-art PtG systems^65,95,96^. The highest reported solar-to-CH_4_ efficiency is 2.41%^95^ and most PEC systems exhibit efficiencies of at least one order of magnitude lower than this.

## Outlook

Solar CH_4_ could eventually replace fossil CH_4_. Further academic and industrial research is, however, necessary to successfully implement solar CH_4_ technologies. The maturity of the solar CH_4_ synthesis approaches discussed in this review vary from scientific proof-of-concept (i.e., biophotocatalytic and homogeneous photoredox systems) to successfully operating small-scale benchtop devices and photoreactors (i.e., photothermal and heterogeneous photoredox systems). Additional research is needed to scale up solar CH_4_ to the industrial scale. Novel and improved photocatalysts and photoreactor designs will be necessary to enable the continuous production of solar CH_4_ with high conversion efficiency, selectivity, and conversion, while simultaneously minimizing the associated raw material, processing, and manufacturing costs. Only then will large-scale or modular solar CH_4_ refineries be readily and rapidly deployable across existing power generation, transportation, heating, and industrial sectors. Solar CH_4_ technology could relatively seamlessly integrate into existing energy infrastructure (such as storage, pipeline, and distribution facilities) at low cost and with minimal impact on the environment and landscape. In this regard, solar CH_4_ technology seems inclined toward public acceptance, providing a strong contribution towards ameliorating global warming and relieving climate change.
